# Targeted Transdermal Delivery of Curcumin for Breast Cancer Prevention

**DOI:** 10.3390/ijerph16244949

**Published:** 2019-12-06

**Authors:** Michele Atlan, Josh Neman

**Affiliations:** 1 Breast Cancer Care & Research Fund, Los Angeles, CA 90036, USA; 2 Department of Neurological Surgery, Los Angeles, CA 90033, USA; yebrahim@usc.edu; 3 Department of Physiology & Neuroscience, Los Angeles, CA 90033, USA; 4 Norris Comprehensive Cancer Center, Los Angeles, CA 90033, USA; 5 Keck School of Medicine of University of Southern California, Los Angeles, CA 90033, USA

**Keywords:** breast cancer prevention, curcumin, bioavailability, transdermal delivery

## Abstract

N.B. This manuscript is based on the research concept submitted to the “Global Challenge to Prevent Breast Cancer” idea showcase and competition, launched in 2018 by the California Breast Cancer Research Program (CBCRP), which was subsequently selected for publication. The hypothesis, methods, and discussion put forth here are thus proposed concept studies, which could eventually be elucidated in the future. Curcumin is an herbal supplement, shown in preclinical studies to have antioxidant, anti-inflammatory, and antitumoral properties that we believe can be harnessed for breast cancer prevention. However, due to its poor absorption when consumed orally, curcumin’s anticancer effects have not yet been exploited to their full therapeutic potential. Incorporating existing research that focuses on the optimization of curcumin’s bioavailability and the latest transdermal delivery technology, we propose, below, a hypothetical in vivo study to test whether a targeted daily dose of bioavailable curcumin has a cytotoxic effect on cancer cells, potentially reducing the incidence of breast cancer over time. Our ultimate objective is to adopt innovative methods to create curcumin-infused bio-textiles offering transdermal, targeted drug delivery, simply through contact with the skin. We would use this fabric to create disposable bra inserts for an effortless, daily breast cancer prevention regimen for healthy women. It would be essential that the cost of these inserts remain reasonable, but if successful, curcumin is readily available, affordable and non-toxic, and could thus be a preventive measure that would be beneficial for women from all socio-economic backgrounds.

## 1. Introduction

Currently, one out of eight women in the United States will contract some form of breast cancer in her lifetime [1]. Despite advances in screening, research, and treatment, breast cancer incidence continues to rise, and breast cancer mortality rates remain far too high—especially among non-white women. To eventually eliminate deaths from breast cancer, focusing on preventive measures is crucial; however, today, primary prevention of breast cancer constitutes less than 10% of the research being funded [2]. Because the genetics, environmental exacerbators, and biological pathways leading to the disease are multivariate and incredibly complex, there are no simple panaceas. Preventive initiatives taken by healthy women must be not only non-toxic and inexpensive, but also practical and easy to comply with.

In 2018, the California Breast Cancer Research Program launched a Global Challenge competition and asked both researchers and advocates to submit bold, out-of-the-box research ideas for preventing breast cancer. As a finalist in the advocate category, we were invited to write up and expand on the contest submission. Our premise was to seek a more natural, plant-based solution to breast cancer prevention.

An increasing number of studies are proposing that certain phytonutrients can be a powerful addition to other breast cancer prevention methods. However, even if we are able to exploit their disease fighting properties, we would need to develop efficient delivery systems in order to take advantage of their treatment benefits. Curcumin is one natural product that has been of particular interest to researchers, and we will be exploring its therapeutic potential in terms of breast cancer prevention, as well as discussing a novel delivery system to target and improve its efficacy.

Curcumin is the primary active ingredient of the Indian spice turmeric (*Curcuma longa*), as well as the source of its rich golden color. For thousands of years, turmeric has been used medicinally as a treatment for inflammatory disorders, particularly in the Ayurvedic tradition [3]. It is in fact made up of three different curcuminoids: curcumin (diferuloylmethane), demethoxycurcumin, and biscemethoxycurcumin, as well as a complex of volatile oils, proteins, resins, and sugars [4]. However, it is the curcumin molecule that has attracted the most attention from scientists in recent decades. Curcumin from turmeric is easily sourced, extremely affordable, and clinically proven to be safe in doses up to 8 g/day [5]. Early research, using cell cultures and mouse models, has focused on its established antioxidant and anti-inflammatory properties, as well as its possible anticarcinogenic capacities. Curcumin is believed to act pleiotropically on multiple molecular and cellular pathways, and this could explain its potential to combat multifactorial diseases such as cancer.

Review of evidence about the clinical validity of curcumin is complex because its therapeutic rationale has stirred up controversy. Despite the fact that in 2017, there were already over 15,000 manuscripts published examining the biological properties of curcumin, the results of this large pool of research have been mainly disregarded since curcumin’s beneficial effects have not yet been proven in a randomized placebo-controlled clinical trial. Nelson and colleagues published a mini-perspective on “The Essential Medicinal Chemistry of Curcumin” and posit that inconclusive results have given curcumin “the label of pharmacodynamically fierce (hits many targets) yet pharmacokinetically feeble (does not get to its targets)” [6]. This article was rebutted by Dutch researcher Michal Heger on behalf of 13 correspondents, who cites 49 double-blind clinical trials, of which 17 showed efficacy. Furthermore, he states that “the binding behavior of curcumin to multiple molecular targets is associated with modulation rather than outright inhibition” [7]. We understand that there is not yet a conclusive answer to this debate, but we believe that pursuing novel targeted delivery methods might further elucidate whether curcumin could indeed have salutary prospects.

Oxygen-derived free radicals are generated as a normal part of the cellular aerobic process and one category of these, reactive oxygen species (ROS), serves as a necessary intermediate in many enzyme reactions. But despite this helpful role and because of their reactive nature, increased levels of ROS can be highly toxic to normal cells, damaging and interfering with the repair mechanisms of their DNA. In vitro studies have demonstrated curcumin’s ability to impair the generation of ROS, preventing, among other things, its activation of the oncogenic effects of activator protein-1 (AP-1), a transcription factor that controls a number of cellular processes including proliferation and apoptosis [8,9].

As far as anti-inflammation is concerned, topical curcumin has historically been used to reduce wound-healing time and reduce scarring, as well as serving as an antimicrobial agent. Curcumin’s anti-inflammatory actions are most likely linked to its ability to inhibit the expression of specific proinflammatory cytokines and enzymes, high levels of which have been shown to contribute to cancer development [10]. For example, a pilot study of curcumin for women with obesity and a higher risk for breast cancer is currently seeking to determine if different doses of curcumin will modulate proinflammatory biomarkers in this population in a randomized clinical trial [11]. Inflammation in the cancer micro-environment not only aids in the formation of tumors, but also contributes to their progression and metastasis.

Preclinical cancer research has indicated that curcumin hinders tumorigenesis in a number of cancer types by inhibiting cell proliferation, inducing apoptosis and preventing metastasis. In vitro and in vivo studies specific to breast cancer have explored curcumin’s effect on a variety of pathways and cellular activities such as its inhibition of telomerase activity in the MCF-7 breast cancer cell line [12]. It has an anti-proliferative effect on MDA-MB-231 and BT-483 breast cancer cell lines, in a time- and dose-dependent manner, by down-regulating the genes that induce NFκB, a type of transcription factor that influences cell proliferation and cell survival [13,14]. The apoptotic influence of curcumin was also tested on tumor suppressor and hormone receptor dysregulation in breast cancer cells, to positive results [15]. Curcumin has been evaluated in combination with other phytonutrients as well, both in vitro and in vivo. A 2017 study combined curcumin with another natural product, arabinogalactan, to see if, together, they would decrease breast cancer cell growth without affecting normal cells. The combination was shown to induce apoptosis by increasing ROS levels in human breast cancer cell lines, and to inhibit tumor growth through the regulation of p53 and the reduction of Ki67, in murine breast cancer cells implanted into BALB/c mice [16]. Despite all these promising preclinical results, curcumin’s antitumoral effects have yet to be exploited to their full therapeutic potential in human clinical trials due to its low water solubility and poor bioavailability. When administered orally, its rapid metabolism in the intestine and hepatic system unfortunately results in 60% to 70% of the compound being eliminated unmetabolized in the feces [17]. In order to overcome these obstacles, researchers are turning to nanotechnology, which we will touch on below.

In this commentary, we will be evaluating research on the current methods for rendering curcumin water-soluble and bioavailable to see if we can determine the safest, most natural and most efficient form of functional curcumin to propose for breast cancer prevention. In addition, we hypothesize that transdermal delivery of the bioavailable curcumin directly to the breast area could, potentially, not only increase its potency, but also pinpoint its focus [18]. With the collaboration of Dr. Josh Neman, we imagined a preclinical experiment that could test this hypothesis in mouse models. To conclude, we will discuss the modalities of eventually incorporating bioavailable transdermal curcumin into smart textiles for the production of disposable bra inserts. If successful, this would enable us to create an innovative form of safe, natural, and “wearable” breast cancer prevention for testing in future clinical trials.

## 2. Approaches and Methods

To address the limitations of curcumin in terms of its inherent instability and poor bioavailability, studies exploring the production of synthesized curcumin analogs have shown promise for increased apoptosis in breast cancer cells [19]. Numerous curcumin analogs have been tested by creating chemical modifications of the original molecule or synthesizing new curcumin compounds in the lab with varying results [20]. However, for the purposes of the present hypothesis to evaluate targeted transdermal delivery of curcumin, we would opt for other bioavailability techniques since these analogs would need to be assessed as entirely new chemical compounds [6].

The initial drawback of curcumin to be overcome is its hydrophobic nature. This has been solved with the advent of curcumin nanoencapsulation. Nanoparticle technology shrinks and coats curcumin molecules, making them soluble in water. Nanocurcumin prepared by a wet-milling process retains the same chemical structure as curcumin, but renders it water soluble due to the reduced size of its particles [21]. In a 2013 study, poly(lactic-*co*-glycolic acid) (PLGA) nanoparticles were shown to induce a cell cycle arrest on MCF7 breast cancer cells by releasing curcumin in the cytoplasm, preventing proliferation in a time- and dose-dependent manner [22]. In the interests of developing a hydrophilic curcumin molecule without the use of potentially toxic chemicals, a 2017 study was able to prepare water-soluble curcuminoids from turmeric powder by using steviol glucosides, the natural, non-toxic compounds of the stevia plant [23].

In terms of bioavailability, a technique being studied is the formulation of a curcumin-loaded liposomal hydrogel that can provide a prolonged release in target tissues [24]. In order to penetrate the skin barrier for transdermal delivery, however, standard liposome-based drug carriers are ineffective. The carrier must be able to traverse the skin physically intact and to do so, it must be elastic enough to squeeze through microconduits in the skin. Deformable liposomes called Transferosomes^®^, first introduced by Cevc and Blume in 1992, present an ingenious solution to this predicament [25]. Transferosomes^®^ are bilayer vesicles, loaded with a specific drug or active ingredient, that can cross the skin barrier, reach deep below the application site and release the drug molecules slowly at the target site. They can squeeze through pores 5–10 times smaller than their own diameter. Passive delivery through the skin is rendered possible through osmotic gradient, with the Transferosomes^®^ being drawn from the drier skin surface down to the more hydrated deep skin dermis where they can then enter the lymphatic and systemic circulation [26,27]. We therefore believe that a curcumin-loaded Transfersomal hydrogel would be an optimal delivery system, for testing in vivo, to evaluate the potential benefits of targeted transdermal curcumin for breast cancer prevention.

To ascertain if this localized transdermal delivery of curcumin has an effect on breast tumor prevention and initiation, we suggest the following in vivo studies. First, we will utilize transgenic breast cancer mouse model MMTV-PyMT, which is used to study mammary tumor progression and metastasis. Specifically, MMTV-LTR is used to drive expression of the mammary-gland-specific polyomavirus middle T-antigen. As a result, this spontaneous breast cancer mouse model leads to rapid development of highly metastatic tumors. PyMT mice will be randomized into a control group (*n* = 12) and a curcumin-treated experimental group (*n* = 12). The experimental group would receive daily, topically applied transdermal hydrogel containing curcumin (30 mg/kg) to the mammary fat pad area, covered by an adhesive gauze to prevent the mice licking or scratching it off; control group would get topical hydrogel alone. We would subsequently monitor for tumor latency, initiation, growth, and size. This would permit us to see if transdermal curcumin has a chemopreventive effect on incidence, as well as a cancer fighting potential. Time to appearance of first tumor would be compared between both groups, and we would estimate changes in survival using a Kaplan–Meier Curve. Weekly calipers or Micro-PET scanning techniques would be used for volumetric measurement and tumor growth, respectively. Potential toxicity and deleterious effect on normal breast cells, if any, would be noted. At end-point, animals will be euthanized and tissue staining and histology will be conducted testing for tumor pathology. This would include Ki67 proliferative marker, CD31 endothelial vascular marker, and CD4/CD8 for immune detection. Importantly, we would check ensuing levels of the NFκB transcription factor and the inflammatory cytokine tumor necrosis factor alpha (TNF-α), both of which are important biomarkers for all subtypes of breast cancer.

Subsequently, in order to test the effect of curcumin on human breast cancers and to model tumor growth and metastases, we would test the effect of topical curcumin on PDX mice [28]. Using humanized mice, it would be of interest to test curcumin’s cytotoxic impact on ER/PR+, Her2+, and Triple Negative breast cancer subtypes to see if there are any differences in effects or outcomes. Specifically, patient-derived breast cancer cells (SKBR3, MDA-MB231, and MCF7) expressing firefly luciferase would be implanted (10,000 tumor cells/10uL) in the mouse mammary fat pads, permitting us to monitor the growth and potential spread of tumors through bioluminescent imaging. We would randomize the mice into control (*n* = 12) and experimental (*n* = 12) groups and apply the curcumin hydrogel to the experimental group as previously described. In order to determine the efficacy of topical curcumin, bioluminescence analysis (BLI) of tumors would be conducted weekly to determine tumor growth and metastasis, and survival. Tissue analysis would be conducted postmortem on both animal groups as described above.

## 3. Expected Results and Discussion

### Theoretical Results

These proposed mouse studies have not been conducted; therefore, our results, by necessity, remain hypothetical. Based on our hypothesis, we predict observing a delay to progression of tumorigenesis, as well as a reduction in the numbers and size of tumor growth in the topical curcumin experimental arm compared to control for both the transgenic and xenograft in vivo studies. Though curcumin has been proposed to affect a variety of tumorigenic cellular processes and signaling pathways, we would focus on measuring the levels of NFκB and TNF-α to see if their anticipated reduction is associated with a reduction in proliferation and an increase in apoptosis of tumor cells.

The proinflammatory transcription factors in the NFκB family regulate more than 500 genes that mediate cell survival, stimulate proliferation, block apoptosis, and stimulate angiogenesis and metastasis in breast cancer cells. In addition, activation of the NFκB signaling pathway can induce gene expressions that create an inflammatory microenvironment conducive to carcinogenesis [29,30]. Curcumin has been shown to down-regulate the expression of NFκB [31], so if our animal studies result in a reduction of its levels, as hypothesized, this could give us an indication of its potential cytotoxic impact on breast cancer cells.

The correlation of inflammatory biomarker TNF-α with increased tumor size, stage, and lymph node metastasis in breast cancer is verified, and the expression of TNF-α is also presumed to be linked to NFκB activation [32]. Anticipated results in our mouse model study showing a reduction of TNF-α levels would further indicate a positive role for targeted transdermal curcumin as a convincing anticarcinogenic agent for use in breast cancer prevention.

## 4. Conclusions

If the anticipated results from our experimental studies show transdermal curcumin targeted to the breast area as a possible cytotoxic agent against existing breast cancer cells, we speculate that it might also prevent the subsequent formation of tumors. Creating a salubrious breast environment by modulating inflammatory biomarkers, limiting dangerous ROS damage to normal breast cells, and eliminating incipient abnormal cells before they proliferate and become cancerous should, in theory, enable us to reduce the future incidence of breast cancer.

Despite current statistics showing that one out of eight women in the United States will contract breast cancer in her lifetime, young, healthy women with no family history of the disease are unlikely to actively engage in preventive measures, especially if they are onerous. The importance of breast cancer prevention is indisputable, but how does one encourage healthy young women to willingly partake in it? More importantly, what safe, inexpensive, and appealing measures can be provided? Inventing a simple, daily tool that would not only maximize their breasts’ health, but also enhance their appearance using natural ingredients, could potentially convince women of all ages to engage in breast cancer prevention. Our ultimate goal is to create disposable bra inserts made of curcumin-loaded fibers and containing natural, skin-enhancing supplements to incorporate breast health into women’s daily beauty regimens.

Recent advances in bio-textiles are seeking ways for active ingredients to traverse the skin barrier, without the use of adhesive patches. The fabric would simply need to be in contact with the skin. The production of wearable textiles with a controlled slow-release delivery system of active ingredients is already a reality. Among others, the company Textile-Based Delivery, Inc. (TexDel), founded by CEO Jordan Schindler in 2012, has patented a technique for embedding drugs and neutroceuticals directly into individual yarn fibers [33]. The content and delivery rates can be tailored to ensure that a constant, safe level of active ingredients is administered daily. Fiber-embedded medicine is absorbed during contact with the skin, responding to precise skin temperature and moisture levels [34]. Using similar techniques, we could create inexpensive, disposable bra liners infused with curcumin (as well as natural skin supplements such as collagen, biotin, green tea, or honey), that can be adapted to all existing bra brands, sizes, and shapes. By simply putting on a bra, which is usually worn for at least eight hours a day, women would be receiving health benefits without any active effort on their part.

Because these ingredients are non-toxic and easily sourced, and with the incorporation of transfersomal hydrogel into the fabric for its excellent skin penetrating properties, this could be an excellent tool to be tested in future clinical trials for breast cancer prevention in healthy women. By initially testing them on women at a higher risk for breast cancer, such as obese women or those with dense breast tissue, we could see if there is a reduction in their inflammatory biomarkers over time. Breast cancer prevention trials, by necessity, require long-term follow-up. However, the cosmetic aspect of improving skin quality and appearance would help to ensure compliance in the study population.

Critically, the inserts would eventually need to be inexpensive enough for use by women from all socio-economic backgrounds. Collaborating with industry partners, such as, for example, female-run bra companies, might be an effective way to produce the inserts, even before clinical trial results become available. They could be initially marketed as a beauty-based product with potential disease prevention benefits to be determined. Many companies donate one product for every unit sold, and donations of inserts to free clinics or women’s health centers would help underserved communities have access to the inserts for a nondiscriminatory impact.

Due to the ever-increasing trend of exploring ecological and organic healthcare solutions, using plant-based ingredients for breast cancer prevention is an attractive concept that researchers have been actively seeking to implement. Unfortunately, due to lack of bioavailability and inefficient delivery methods, most studies examining phytonutrients, such as curcumin, have been inconclusive [33]. Our concept of combining existing research to render curcumin bioavailable with new forms of transdermal delivery systems could enable a breakthrough. By testing the anticancer efficacy of targeted bioavailable curcumin in animal studies, we hope to extrapolate the findings for future human clinical trials, and eventually create a breast cancer prevention method that would be appealing to healthy women of all ages. If we are able to repurpose an affordable, inexpensive natural ingredient to reduce the worldwide incidence of breast cancer, we could truly change our odds in the fight against this pervasive disease.
